# Occasional cooperative breeding in birds and the robustness of comparative analyses concerning the evolution of cooperative breeding

**DOI:** 10.1186/s40851-016-0041-8

**Published:** 2016-03-28

**Authors:** Michael Griesser, Toshitaka N. Suzuki

**Affiliations:** Department of Anthropology, University of Zurich, Winterthurerstrasse 190, 8057 Zurich, Switzerland; Department of Evolutionary Studies of Biosystems, SOKENDAI (The Graduate University for Advanced Studies), Kanagawa, Japan

**Keywords:** Cooperative breeding, Alloparental care, Comparative studies, Interspecific feeding, Incidental observations

## Abstract

Cooperative breeding is a widespread and intense form of cooperation, in which individuals help raise offspring that are not their own. This behaviour is particularly well studied in birds, using both long-term and comparative studies that have provided insights into the evolution of reproductive altruism. In most cooperatively breeding species, helpers are offspring that remain with their parents beyond independency and help in the raising of younger siblings. However, many cooperatively breeding species are poorly studied, and in 152 species, this behaviour only has been observed infrequently (i.e., occasional cooperative breeding). Here we argue that the parental care mode of these 152 species needs to be treated with caution, as factors associated with occasional cooperative breeding may differ from those associated with “regular” cooperative breeding. In most cooperatively breeding species, helpers provide alloparental care at the nests of their parents or close relatives; however, only in one occasionally cooperatively breeding species do offspring remain into the next breeding season with their parents. Accordingly, different factors are likely to be associated with regular and occasional cooperative breeding. The latter behaviour resembles interspecific feeding (i.e., individuals feed offspring of another species), which occurs when birds lose their brood and begin feeding at a nearby nest, or when birds mistakenly feed at another nest. Thus, we advise researchers to exclude occasional cooperative breeders in comparative analyses until their status is clarified, or to categorize them separately or according to the typically observed parental care mode. This approach will increase the robustness of comparative analyses and thereby improve our understanding of factors that drive the evolution of cooperative breeding.

**Electronic supplementary material:**

The online version of this article (doi:10.1186/s40851-016-0041-8) contains supplementary material, which is available to authorized users.

## Introduction

Cooperation among individuals occurs at all levels of biological organisation [1] and understanding the factors that select for cooperation is a fundamental goal of evolutionary biology. Darwin perceived that reproductive cooperation, such as sterile casts in eusocial insects, represented a challenge to his theory of natural selection [2]. Over the past 50 years, research has shown that cooperation often occurs among related individuals [1, 3], providing opportunities for kin selected fitness benefits [4]. One intensive form of cooperation is alloparental care [1, 5–7], in which individuals help raise the offspring of others while often foregoing their own reproduction [8, 9]. This behaviour has evolved in all major vertebrate lineages and is particularly well investigated in birds, using large-scale comparative studies [10–14].

Earlier comparative studies have investigated the association between eco-climatic and life-history factors and the occurrence of cooperative breeding, yielding sometimes contradictory findings [10–13]. While some studies have suggested that cooperative breeding is associated with stable climatic conditions and saturated habitats [10, 12, 15], other studies have indicated that it is associated with unpredictable climatic conditions [11, 16, 17]. These contradictory findings may in part reflect heterogeneity in the quality of data on the parental care mode of birds. To consider this uncertainty, we categorize species in which cooperative breeding has been observed infrequently as “occasional cooperative breeders” (*N* = 152 species, representing 15 % of both cooperatively and occasionally cooperatively breeding species [18, 19]; supplementary material Additional file 1: Table S1). Observe that we categorized the Darwin finches *Geospiza scandens* and *G. fortis* as occasional cooperative breeders, based on a detailed study on helping at the nest in these two species [19] (see below). However, the question of how to categorize the mode of parental care in occasionally cooperatively breeding species remains open, and small numbers of observations may not be sufficient to categorize these species [20].

Here, we review the current understanding of the routes to cooperative breeding, and summarize previously published studies of the family structure of occasional cooperative breeders. We propose that occasional cooperative breeding shows parallels with interspecific feeding (i.e., individuals feed offspring of another species), and thus should not be categorized together with “regular” cooperatively breeding species in comparative studies.

## Routes to cooperative breeding

In nearly all cooperatively breeding species (i.e., 93 % [18, 21, 22]), helpers are offspring that remain associated with their parents until the next breeding season and provide alloparental care at the nest of their parents or close relatives [9, 21, 22]. Field studies have demonstrated that helpers can gain both direct and indirect, kin-selected fitness benefits from providing alloparental care [3, 22, 23]. In some cooperatively breeding species, however, helpers are not related to breeders, but these individuals often have a share in reproduction, the species breeds polygynously or polyandrously (such as ani *Crotphasgus sp.*), or helpers queue for a breeding position [5, 22]. Thus, this route to cooperative breeding is most likely facilitated by direct fitness benefits [23]. Both of these phenomena occur regularly within populations, but the number of pairs that receive alloparental care can vary between 0 and 100 % depending on the species and annual conditions [5, 6]. Also, individuals may express a high flexibility in their parental care contributions in some species and frequently switch roles between breeders and helpers even within a breeding season [24]. Finally, three individuals have been observed feeding at the same nest in few instances in 152 species (labelled occasional cooperative breeding [18]), but the factors selecting for this behaviour remain unclear.

## Occasional cooperative breeding

**Fig. 1:**
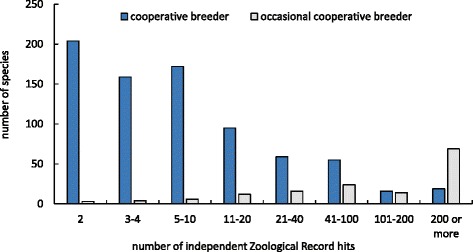
Distribution of the number of independent Zoological Record entries for cooperatively breeding species, including species that are suspected cooperative breeders (data available for *N* = 782 out of 864 species), and occasionally cooperatively breeding species (data available for *N* = 146 out of 152 species). Most cooperatively breeding species have only two independent Zoological Record entries, while most occasionally cooperatively breeding species are well studied

By definition, occasional cooperative breeding occurs rarely, and is thus difficult to investigate and remains poorly understood. This behaviour may occur commonly in some species, but be overlooked in species that are poorly investigated. However, while most cooperatively breeding species are not well studied, (i.e., cooperatively breeding species have a mode of two independent Zoological Record entries; data obtained from [25]; Fig. 1), many occasionally cooperatively breeding species in fact are well studied (mode of independent Zoological Record entries of occasional cooperative breeder = 15; *N* = 69 species have more than 200 independent Zoological Record entries; Spearman rank correlation: *P* < 0.001; Fig. 1). For example, occasional cooperative breeding has been observed in model species, including mute swan *Cygnus olor*, common guillemot *Uria aalge*, blue tit *Parus caeruleus*, white stork *Ciconia ciconia*, common tern *Sterna hirundo* or house sparrow *Passer domesticus*. In these species, “helpers” are most likely unrelated to the breeders, since the offspring do not remain with their parents into the next breeding season, but disperse earlier (see Additional file 2: Table S2 for details) [21, 26–28]. Only in one occasional cooperative breeder, the Verreaux’s eagle owl *Bubo lacteus*, offspring may remain up for to 2 years in the parental territory [29], and thus live in family groups in a manner similar to that of most cooperatively breeding species.

**Fig. 2:**
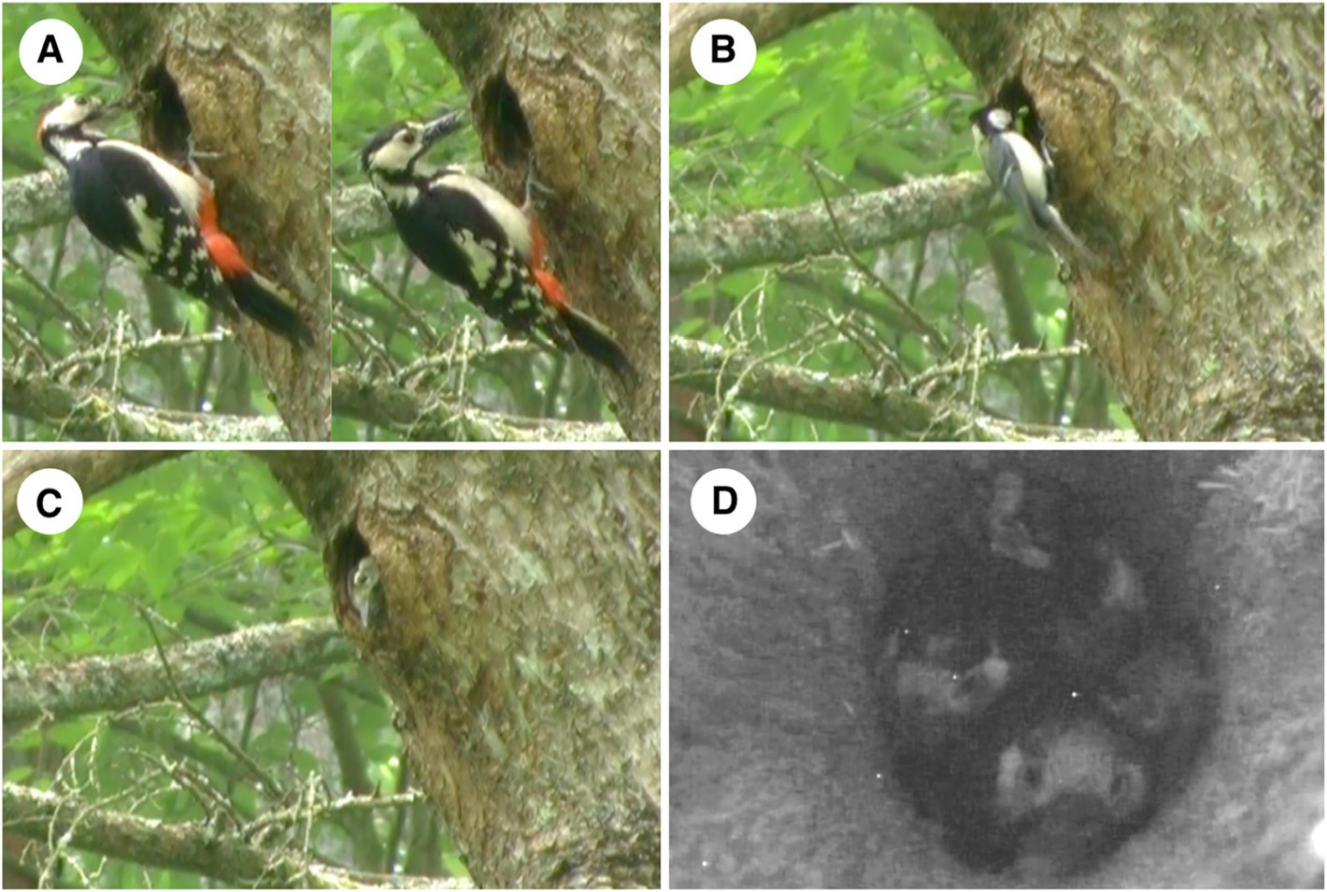
An example of interspecific feeding. During fieldwork on Japanese great tits *Parus minor* in Karuizawa, Nagano Prefecture, Japan (36.33’138’’ N, 138.56’260’’ E) 15-18 June 2015, we observed a nest of a great spotted woodpecker *Dendrocopos major* where in addition to the parents. (**a**), a Japanese great tit male fed the young woodpeckers (**b**). Based on video recordings, the feeding rate of the parents was much lower than the feeding rate by the great tit (feeding rates: male parent: 5.2/hr, female parent: 4.1/hr, great tit male: 17.2/hr, total time assessed: 14.5 hrs). Inspection with an infrared camera confirmed that the cavity contained four great spotted woodpecker nestlings but no great tit nestlings (**c-d**). Moreover, the woodpeckers often displaced the male Japanese great tit from the cavity

A single study on Darwin finches *Geospiza scandens* and *G. fortis* described occasional cooperative breeding in these species in detail [19]. Eleven unpaired adult males were observed to provide alloparental care during a severe drought. However, the helpers were not related to the breeding pair, and many helpers bred earlier on in their lives, excluding that they helped to gain breeding experience. Moreover, none of the females that received help paired up with a helper later on in their lives, and helpers did not seem to gain access to reproduction. Rather, the authors suggested that the alloparental care reflected “misdirected” parental care [19, 30, 31].

## Occasional cooperative breeding and interspecific feeding

Occasional cooperative breeding shows parallels with interspecific feeding (i.e., individuals feed offspring of other species [32]; Figure 2). In both cases, individuals do not feed related offspring, and both behaviours occur rarely. Interspecific feeding has been observed in 51 species (Additional file 2: Table S2; excluding cases where individuals from two species share a nest or egg dumping leads to mixed broods). It has been suggested that the main reason behind interspecific feeding is the loss of the individual’s own brood, leading to those individuals to begin feeding at a nearby nest of another species [30, 32, 33]. Alternatively, parents may have their own nest but accidentally feed at another nest due to the close proximity of the other nest, or in response to the begging calls of nestlings [20, 32].

Interspecific feeding has been documented in a wide range of bird species (Table 1, Additional file 2: Table S2; see also [32]), suggesting that individuals may feed the young of conspecifics (i.e., engage in occasional cooperative breeding) for the same reasons as individuals engage in interspecific feeding [19]. Indeed, ten species are known to engage both in occasional cooperative breeding and interspecific feeding (i.e., 19.6 % of all occasional cooperative breeders; Additional file 1: Table S1, Additional file 2: Table S2). Interspecific feeding and occasional cooperative breeding, however, differ in one very critical point: the former is easy to recognize and categorize in the field, but a single observation of three individuals feeding at a nest does not allow categorization. It became quickly evident that the Japanese great tit is not a cooperatively breeding species, but one that may engage in interspecific feeding (Figure 2). If the Japanese great tit male had fed at another nest of Japanese great tits, however, we may have classified this observation as regular or occasional cooperative breeding. The generally used definition of cooperative breeding (an individual helps in the raising of offspring that are not their own, often while foregoing their own reproduction [5, 6, 8, 9]) does not differentiate whether the behaviour occurs within species (i.e. cooperative breeding), or across species (i.e. interspecific feeding). Thus, we propose to adjust the definition of cooperative breeding to: an individual that helps in the raising of *conspecific* offspring that are not their own, often while foregoing their own reproduction.

**Table 1:** Taxonomic overview on the family level over the parental care mode of occasional cooperative breeders and of 9659 bird species, the number of species showing interspecific feeding, the proportion of cooperatively breeding species within all families, and the number of species in each taxonomic family; CB = cooperative breeder (including both family living cooperative breeders and species with unrelated helpers), FAM = family living species, NO-FAM = non-family living species, UN = unknown parental care mode

Taxonomic family	Parental care mode of occasional CB species			Inferred parental care mode of all species				N species engaging in interspecific feeding	Proportion CB species	Total number of species
	FAM	NO-FAM	UN	CB	FAM	NO-FAM	UN			
**Acanthisittidae**	0	0	0	1	0	1	0	0	**0.50**	2
**Acanthizidae**	0	0	0	31	15	10	8	0	**0.48**	64
Accipitridae	3	11	6	15	30	53	139	1	0.06	237
**Aegithalidae**	0	0	0	5	0	5	0	1	**0.50**	10
Aegithinidae	0	0	0	0	0	0	4	0	0.00	4
Aegothelidae	0	0	0	0	0	1	7	0	0.00	8
Alaudidae	0	2	2	2	6	35	47	0	0.02	90
Alcedinidae	0	2	1	15	2	13	65	0	0.16	95
Alcidae	0	1	0	0	0	18	4	0	0.00	22
Anatidae	4	0	0	0	32	61	61	0	0.00	154
Anhimidae	0	0	0	0	1	2	0	0	0.00	3
Anhingidae	0	0	0	0	0	4	0	0	0.00	4
**Anseranatidae**	0	0	0	1	0	0	0	0	**1.00**	1
Apodidae	0	0	0	12	0	24	62	0	0.12	98
Apterygidae	0	0	0	1	0	3	1	0	0.20	5
Aramidae	0	0	0	0	1	0	0	0	0.00	1
Ardeidae	0	0	0	0	5	42	15	0	0.00	62
**Artamidae**	0	0	0	9	0	2	0	0	**0.82**	11
Atrichornithidae	0	0	0	0	1	1	0	0	0.00	2
Balaenicipitidae	0	0	0	0	0	1	0	0	0.00	1
Bombycillidae	0	0	0	0	0	4	4	0	0.00	8
Brachypteraciidae	0	0	0	0	1	0	4	0	0.00	5
Bucconidae	0	0	0	5	0	4	24	0	0.15	33
**Bucerotidae**	1	0	0	21	8	12	13	0	**0.39**	54
**Bucorvidae**	0	0	0	2	0	0	0	0	**1.00**	2
Burhinidae	0	0	0	0	2	4	3	0	0.00	9
Callaeatidae	0	0	0	0	0	2	0	0	0.00	2
Campephagidae	1	0	0	5	5	19	55	0	0.06	84
Caprimulgidae	0	0	1	0	4	10	71	0	0.00	85
Cardinalidae	0	1	0	13	6	21	18	2	0.22	58
Cariamidae	0	0	0	0	2	0	0	0	0.00	2
Casuariidae	0	0	0	0	1	2	0	0	0.00	3
Cathartidae	0	0	0	0	3	1	3	0	0.00	7
Certhiidae	0	0	0	0	2	4	1	0	0.00	7
Charadriidae	0	0	2	1	8	33	23	0	0.02	65
Chionidae	0	0	0	0	1	1	1	0	0.00	3
Chloropseidae	0	0	0	0	0	2	6	0	0.00	8
Ciconiidae	0	0	2	0	2	9	8	0	0.00	19
Cinclidae	0	0	0	0	0	2	3	0	0.00	5
**Cinclosomatidae**	0	0	0	3	0	0	2	0	**0.60**	5
Cisticolidae	0	0	0	14	48	27	29	1	0.12	118
**Climacteridae**	0	0	0	5	1	1	0	0	**0.71**	7
Cnemophilidae	0	0	0	0	0	3	0	0	0.00	3
Coerebidae	0	0	0	0	0	0	1	0	0.00	1
**Coliidae**	0	0	0	6	0	0	0	0	**1.00**	6
**Colluricinclidae**	0	0	0	6	0	0	7	0	**0.46**	13
Columbidae	0	0	0	0	3	276	25	0	0.00	304
Conopophagidae	0	0	0	0	0	8	0	0	0.00	8
Coraciidae	0	0	1	1	0	3	8	0	0.08	12
**Corcoracidae**	0	0	0	2	0	0	0	0	**1.00**	2
**Corvidae**	1	0	0	48	35	31	5	0	**0.40**	119
Cotingidae	0	0	0	1	5	60	28	0	0.01	94
Cracidae	1	0	0	0	38	6	6	0	0.00	50
**Cracticidae**	0	0	0	7	0	1	4	0	**0.58**	12
Cuculidae	0	2	1	4	4	87	47	0	0.03	142
Dasyornithidae	0	0	0	0	0	3	0	0	0.00	3
Dendrocolaptidae	0	0	0	0	5	7	34	0	0.00	46
Dicaeidae	0	0	0	0	4	14	26	0	0.00	44
Dicruridae	0	0	0	1	4	7	12	0	0.04	24
Diomedeidae	0	0	0	1	0	13	0	0	0.07	14
Dromadidae	0	0	0	0	0	1	0	0	0.00	1
Dromaiidae	0	0	0	0	1	0	0	0	0.00	1
**Dulidae**	0	0	0	1	0	0	0	0	**1.00**	1
Emberizidae	0	5	0	7	40	200	85	6	0.02	332
Estrildidae	1	0	0	0	26	89	23	0	0.00	138
Eupetidae	0	0	0	0	1	1	7	0	0.00	9
Eurylaimidae	0	0	0	3	0	3	9	0	0.20	15
Eurypygidae	0	0	0	0	0	1	0	0	0.00	1
Falconidae	0	5	1	15	3	14	31	0	0.24	63
**Falcunculidae**	0	0	0	1	0	0	1	0	**0.50**	2
Formicariidae	0	0	0	0	1	1	59	0	0.00	61
Fregatidae	0	0	0	0	2	3	0	0	0.00	5
Fringillidae	1	6	1	3	30	89	37	3	0.02	159
Furnariidae	0	0	0	8	2	16	209	0	0.03	235
**Galbulidae**	0	0	0	18	0	0	0	0	**1.00**	18
Gaviidae	0	0	0	0	0	5	0	1	0.00	5
Glareolidae	0	0	0	0	3	7	7	0	0.00	17
Gruidae	0	0	0	0	13	1	1	0	0.00	15
Haematopodidae	1	0	0	1	5	3	1	0	0.10	10
Heliornithidae	0	0	0	0	0	2	1	0	0.00	3
Hemiprocnidae	0	0	0	0	0	0	4	0	0.00	4
Hirundinidae	0	6	0	0	3	65	19	1	0.00	87
Hydrobatidae	0	0	0	0	0	10	10	0	0.00	20
Ibidorhynchidae	0	0	0	0	0	0	1	0	0.00	1
Icteridae	0	1	0	11	26	62	12	0	0.10	111
Indicatoridae	0	0	0	0	0	17	0	0	0.00	17
Irenidae	0	0	0	0	0	1	1	0	0.00	2
Jacanidae	0	0	0	0	2	2	4	0	0.00	8
Laniidae	0	1	0	6	8	16	1	0	0.19	31
Laridae	3	0	1	0	18	28	51	1	0.00	97
Leptosomidae	0	0	0	0	0	1	0	0	0.00	1
Machaerirhynchidae	0	0	0	0	0	0	2	0	0.00	2
Malaconotidae	1	0	0	7	14	27	6	0	0.13	54
**Maluridae**	0	0	0	28	0	0	0	0	**1.00**	28
Megapodiidae	0	0	0	0	0	4	15	0	0.00	19
Melanocharitidae	0	0	0	0	1	4	7	0	0.00	12
Meliphagidae	1	5	0	21	13	89	54	0	0.12	177
Menuridae	0	0	0	0	2	0	0	0	0.00	2
**Meropidae**	0	0	0	20	0	0	6	0	**0.77**	26
**Mesitornithidae**	0	0	0	2	0	1	0	0	**0.67**	3
Mimidae	0	0	0	7	8	4	16	2	0.20	35
Momotidae	0	0	0	0	0	0	9	0	0.00	9
Monarchidae	1	0	0	6	16	23	51	0	0.06	96
Motacillidae	0	4	1	0	10	32	23	1	0.00	65
Muscicapidae	0	7	0	26	24	55	179	3	0.09	284
Musophagidae	0	0	0	5	1	13	4	0	0.22	23
Nectariniidae	0	0	1	4	5	48	67	1	0.03	124
**Neosittidae**	0	0	0	2	0	0	0	0	**1.00**	2
Numididae	0	0	0	0	5	1	0	0	0.00	6
Nyctibiidae	0	0	0	0	0	0	7	0	0.00	7
Odontophoridae	0	0	0	0	21	10	0	0	0.00	31
**Opisthocomidae**	0	0	0	1	0	0	0	0	**1.00**	1
Oriolidae	0	0	1	2	1	15	12	0	0.07	30
Orthonychidae	0	0	0	0	1	1	3	0	0.00	5
Otididae	0	0	0	0	10	5	10	0	0.00	25
Pachycephalidae	0	0	0	1	1	0	36	0	0.03	38
Paradisaeidae	0	0	0	0	0	6	34	0	0.00	40
Pardalotidae	0	0	0	1	0	3	0	0	0.25	4
Paridae	1	2	0	14	10	29	4	3	0.25	57
Parulidae	0	4	1	0	7	60	45	2	0.00	112
Passeridae	0	0	0	11	2	24	10	1	0.23	47
Pedionomidae	0	0	0	0	1	0	0	0	0.00	1
Pelecanidae	0	0	0	0	1	3	4	0	0.00	8
Pelecanoididae	0	0	0	0	0	4	0	0	0.00	4
**Petroicidae**	0	1	0	12	0	6	25	1	**0.28**	43
Peucedramidae	0	0	0	0	1	0	0	0	0.00	1
Phaethontidae	0	0	0	0	0	3	0	0	0.00	3
Phalacrocoracidae	0	1	0	0	1	34	1	0	0.00	36
Phasianidae	0	0	0	2	70	77	26	0	0.01	175
Philepittidae	0	0	0	0	0	0	4	0	0.00	4
Phoenicopteridae	0	0	0	0	0	5	0	0	0.00	5
**Phoeniculidae**	0	0	0	5	1	2	0	0	**0.63**	8
Picathartidae	0	0	0	0	0	2	0	0	0.00	2
Picidae	2	2	3	18	44	31	123	3	0.08	216
Pipridae	0	0	0	0	2	24	27	0	0.00	53
Pittidae	0	0	0	0	0	2	29	0	0.00	31
**Pityriaseidae**	0	0	0	1	0	0	0	0	**1.00**	1
**Platysteiridae**	0	0	0	13	12	5	0	0	**0.43**	30
Ploceidae	0	1	1	9	9	57	31	0	0.08	106
Podargidae	0	0	0	0	2	1	11	0	0.00	14
Podicipedidae	5	4	0	0	5	14	0	0	0.00	19
Polioptilidae	0	1	0	0	1	4	9	1	0.00	14
**Pomatostomidae**	0	0	0	5	0	0	0	0	**1.00**	5
Procellariidae	0	0	0	0	0	64	12	0	0.00	76
Promeropidae	0	0	0	0	1	1	0	0	0.00	2
**Prunellidae**	0	0	0	13	0	0	0	0	**1.00**	13
Psittacidae	1	1	0	22	55	63	206	0	0.06	346
**Psophiidae**	0	0	0	3	0	0	0	0	**1.00**	3
Pteroclididae	0	0	0	0	0	16	0	0	0.00	16
Ptilonorhynchidae	0	0	0	0	1	11	8	0	0.00	20
Pycnonotidae	0	0	0	21	38	39	35	0	0.16	133
Rallidae	0	1	0	19	6	14	93	0	0.14	132
**Ramphastidae**	0	0	0	38	12	21	52	0	**0.31**	123
Recurvirostridae	0	0	0	0	4	1	4	0	0.00	9
Reguliidae	0	0	0	0	0	5	1	0	0.00	6
**Remizidae**	0	0	0	7	0	4	1	0	**0.58**	12
Rhabdornithidae	0	0	0	0	0	3	0	0	0.00	3
**Rheidae**	0	0	0	1	1	0	0	0	**0.50**	2
Rhinocryptidae	0	0	0	0	0	1	28	0	0.00	29
Rhipiduridae	0	0	1	0	1	2	39	0	0.00	42
**Rhynochetidae**	0	0	0	1	0	0	0	0	**1.00**	1
Rostratulidae	0	0	0	0	1	1	0	0	0.00	2
Sapayoaidae	0	0	0	0	0	1	0	0	0.00	1
Scolopacidae	0	0	0	0	1	33	53	0	0.00	87
**Scopidae**	0	0	0	1	0	0	0	0	**1.00**	1
Sittidae	0	0	0	2	5	11	7	2	0.08	25
Spheniscidae	0	1	0	0	0	17	0	1	0.00	17
Steatornithidae	0	0	0	0	0	1	0	0	0.00	1
Stercorariidae	0	1	0	2	0	4	2	0	0.25	8
Strigidae	2	3	0	1	14	27	111	1	0.01	153
Struthionidae	0	0	0	0	1	0	0	0	0.00	1
Sturnidae	1	1	0	18	8	77	6	1	0.17	109
Sulidae	0	0	0	0	1	8	1	0	0.00	10
Sylviidae	0	1	1	21	16	50	177	0	0.08	264
Thamnophilidae	0	0	0	2	143	5	46	0	0.01	196
Thinocoridae	0	0	0	0	0	2	2	0	0.00	4
Thraupidae	0	0	0	32	74	110	51	0	0.12	267
Threskiornithidae	0	0	0	0	3	14	15	0	0.00	32
**Timaliidae**	0	0	0	81	18	130	54	0	**0.29**	283
Tinamidae	0	0	0	0	0	7	39	0	0.00	46
**Todidae**	0	0	0	5	0	0	0	0	**1.00**	5
Trochilidae	0	0	0	0	0	310	8	0	0.00	318
Troglodytidae	3	0	0	17	15	9	35	3	0.22	76
Trogonidae	0	0	0	0	1	0	38	0	0.00	39
Turdidae	1	1	0	5	13	115	32	5	0.03	165
Turnicidae	0	0	0	0	3	10	3	0	0.00	16
Tyrannidae	1	0	0	6	37	56	293	3	0.02	392
Tytonidae	0	0	0	0	0	6	10	0	0.00	16
**Upupidae**	0	0	0	1	0	0	0	0	**1.00**	1
Urocynchramidae	0	0	0	0	0	0	1	0	0.00	1
Vangidae	0	0	0	4	3	5	8	0	0.20	20
Viduidae	0	0	0	0	0	17	0	0	0.00	17
Vireonidae	0	0	0	0	5	15	31	0	0.00	51
Zosteropidae	0	1	0	6	5	49	34	0	0.06	94
Total	37	86	29	864	1257	3654	3884	51	0.09	9659

Families with more than 25 % cooperative breeders are highlighted in bold. Taxonomy follows Jetz et al. [44]. See main text and [21] for definitions of the parental care mode. The inferred parental care mode follows Cockburn [18], updated based on the Handbook of the Birds of the World [45]. Observe that we categorized the Darwin finches *Geospiza scandens* and *G. fortis* as occasional cooperative breeders, based on a detailed study on helping at the nest in these two species [19]

## Occasional cooperative breeding and the evolution of regular cooperative breeding

Historically, observations of occasional cooperative breeding have fuelled a debate on the factors favouring the evolution of cooperative breeding [20, 34]. It has been suggested that the initial evolution of alloparental care may be a non-adaptive response to the begging of nestlings [31]. While this behaviour may provide a first step towards cooperative breeding [34], a number of arguments have been put forward regarding why the behaviour of helpers is modified by natural selection, thus making the behaviour adaptive [34]. Physiological studies showed that helpers in cooperative breeders express higher levels of prolactin than individuals that do not help, and that the prolactin level correlates with the helping effort [35, 36]. Moreover, helpers seem to adjust their feeding effort depending on the need of the nestlings [23, 37–40]. Finally, comparative data have shown that helping behaviour is higher in species in which helping at the nest provides a greater fitness benefit, and helpers in species that exhibit extra-pair mating have a higher degree of kin discrimination [4].

## Conclusions

Interspecific feeding and occasional cooperative breeding have intrigued researchers for decades [30] and stimulated a critical assessment of cooperative breeding [20, 34]. Nevertheless, fitness consequences of both interspecific feeding and occasional cooperative breeding remain unstudied due to the rarity of their occurrence [32]. It thus remains difficult to draw firm conclusions as to whether these behaviours are non-adaptive, or provide fitness benefits to the actor. Cooperative breeding has been shown to be adaptive, helpers respond to the actual need of offspring [37], and most helpers provide care at nests of relatives, offering kin selected fitness benefits [22]. Moreover, family living is a stepping stone for the evolution of cooperative breeding [21, 26, 41, 42], but most occasionally cooperatively breeding species do not live in family groups (Additional file 1: Table S1).

Further studies are needed to assess whether “helpers” in occasional cooperative breeders gain direct fitness benefits from doing so (e.g. by having a share in reproduction through extra pair mating or egg dumping). Until the factors that facilitate cooperative breeding in these species are better understood, it may be misleading to categorize such species as cooperative breeders in comparative studies. Thus, we advise excluding these 152 occasional cooperative breeders from analyses of cooperative breeding until their mode of parental care is critically assessed, or to categorize them separately or according to the typically observed parental care mode. This approach will increase the robustness of comparative analyses and thereby improve our understanding of factors that drive the evolution of cooperative breeding.

## Ethical statements

Our field work was approved by the Animal Care and Use Committees at SOKENDAI (The Graduate University for Advanced Studies) and the Forestry Agency of Japan. Our work adhered to the Guidelines for the Use of Animals in Research of the Animal Behavior Society/Association for the Study of Animal Behaviour.

## Additional files

Additional file 1: List of species that are categorized as occasional cooperatively breeding species. (DOCX 45 kb) Additional file 2: List of species that have been observed to engage in misdirected parental care (i.e., feeding young at the nest of another species), excluding cases where species shared nests. (DOCX 30 kb)
