# Supplementary material for: Occasional cooperative breeding in birds and the robustness of comparative analyses concerning the evolution of cooperative breeding
Source: Zoological Lett. 2016 Mar 28;2:7. doi: 10.1186/s40851-016-0041-8 (PMC4810505; doi:10.1186/s40851-016-0041-8)
Supplement: Additional file 1: — List of species that are categorized as occasional cooperatively breeding species. (DOCX 45 kb) [file 40851_2016_41_MOESM1_ESM.docx]

Supplement Table 1. List of species that are categorized as occasional cooperatively breeding species (sorted by taxonomic family), following Cockburn [[1](#_ENREF_1)], and including Darwin finches *Geospiza scandens* and *G. fortis* based on a detailed study on helping at the nest in these two species [[2](#_ENREF_2)]. Taxonomy follows Jetz et al. [[3](#_ENREF_3)], parental care mode and the time offspring remain with their parents beyond independency (family time) follows Drobniak et al. [[4](#_ENREF_4)]. Non-family-living species where offspring disperse soon after independency (no_fam), family-living species where offspring remain well beyond independency with their parents (family), cooperatively breeding, family-living species where offspring remain with their parents and help raising younger siblings (coop_family). Number of Zoological Record entries are obtained from Valcu et al. [[5](#_ENREF_5)].

| scientific name | common name | taxonomic family | engage in misdirected parental care | parental care mode | family time (in days) | number of Zoological Record entries |
| --- | --- | --- | --- | --- | --- | --- |
| Accipiter_cooperii | Cooper's Hawk | Accipitridae | no | no_fam | 20 | 259 |
| Accipiter_nisus | Eurasian Sparrowhawk | Accipitridae | no | unknown | unknown | 1143 |
| Aquila_chrysaetos | Golden Eagle | Accipitridae | no | family | 60 | 1569 |
| Aquila_fasciatus | Bonelli's Eagle | Accipitridae | no | unknown | unknown | 322 |
| Aquila_pomarina | Lesser Spotted Eagle | Accipitridae | no | unknown | unknown | 371 |
| Buteo_augur | Augur Buzzard | Accipitridae | no | no_fam | unknown | 17 |
| Buteo_jamaicensis | Red-tailed Hawk | Accipitridae | no | family | 140 | 732 |
| Buteo_lineatus | Red-shouldered Hawk | Accipitridae | no | unknown | unknown | 245 |
| Buteo_regalis | Ferruginous Hawk | Accipitridae | no | no_fam | 14 | 261 |
| Circus_aeruginosus | Western Marsh-harrier | Accipitridae | no | no_fam | unknown | 714 |
| Circus_cyaneus | Northern Harrier | Accipitridae | no | no_fam | 0 | 828 |
| Circus_maurus | Black Harrier | Accipitridae | no | no_fam | unknown | 29 |
| Circus_pygargus | Montagu's Harrier | Accipitridae | no | no_fam | 0 | 578 |
| Haliaeetus_albicilla | White-tailed Eagle | Accipitridae | no | unknown | unknown | 952 |
| Haliaeetus_leucocephalus | Bald Eagle | Accipitridae | no | no_fam | 35 | 1406 |
| Milvus_milvus | Red Kite | Accipitridae | no | unknown | unknown | 675 |
| Neophron_percnopterus | Egyptian Vulture | Accipitridae | no | family | unknown | 407 |
| Pandion_haliaetus | Osprey | Accipitridae | no | no_fam | 0 | 1270 |
| Pernis_apivorus | European Honey-buzzard | Accipitridae | no | no_fam | unknown | 564 |
| Rostrhamus_sociabilis | Snail Kite | Accipitridae | no | no_fam | 0 | 165 |
| Calandrella_rufescens | Lesser Short-toed Lark | Alaudidae | no | unknown | unknown | 63 |
| Eremophila_alpestris | Horned Lark | Alaudidae | no | no_fam | 16 | 248 |
| Galerida_cristata | Crested Lark | Alaudidae | no | no_fam | unknown | 200 |
| Melanocorypha_calandra | Calandra Lark | Alaudidae | no | unknown | unknown | 71 |
| Alcedo_atthis | Common Kingfisher | Alcedinidae | no | unknown | unknown | 501 |
| Megaceryle_alcyon | Belted Kingfisher | Alcedinidae | no | no_fam | 0 | 30 |
| Todiramphus_chloris | Collared Kingfisher | Alcedinidae | no | no_fam | unknown | unknown |
| Uria_aalge | Common Guillemot | Alcidae | no | no_fam | 0 | 1182 |
| Anser_albifrons | Greater White-fronted Goose | Anatidae | no | family | unknown | 763 |
| Anser_anser | Greylag Goose | Anatidae | no | family | unknown | 1064 |
| Branta_sandvicensis | Hawaiian Goose | Anatidae | no | family | unknown | 99 |
| Cygnus_olor | Mute Swan | Anatidae | no | family | unknown | 1362 |
| Tockus_alboterminatus | Crowned Hornbill | Bucerotidae | no | family | 210 | 19 |
| Coracina_novaehollandiae | Black-faced Cuckooshrike | Campephagidae | no | family | 338 | 36 |
| Caprimulgus_europaeus | Eurasian Nightjar | Caprimulgidae | no | unknown | unknown | 373 |
| Cardinalis_cardinalis | Northern Cardinal | Cardinalidae | yes | no_fam | 0 | 232 |
| Charadrius_dubius | Little Ringed Plover | Charadriidae | no | unknown | unknown | 446 |
| Charadrius_hiaticula | Common Ringed Plover | Charadriidae | no | unknown | unknown | 470 |
| Anastomus_oscitans | Asian Openbill | Ciconiidae | no | unknown | unknown | 60 |
| Ciconia_ciconia | White Stork | Ciconiidae | no | unknown | unknown | 2352 |
| Coracias_garrulus | European Roller | Coraciidae | no | unknown | unknown | 243 |
| Cyanocitta_cristata | Blue Jay | Corvidae | no | family | 120 | 334 |
| Ortalis_vetula | Plain Chachalaca | Cracidae | no | family | 120 | 39 |
| Coccycua_pumila | Dwarf Cuckoo | Cuculidae | no | no_fam | unknown | unknown |
| Coccyzus_americanus | Yellow-billed Cuckoo | Cuculidae | no | unknown | unknown | 156 |
| Coccyzus_melacoryphus | Dark-billed Cuckoo | Cuculidae | no | no_fam | unknown | 6 |
| Ammodramus_henslowii | Henslow's Sparrow | Emberizidae | no | no_fam | unknown | 94 |
| Geospiza_fortis | Medium Ground-finch | Emberizidae | no | no_fam | 7 | 89 |
| Geospiza_scandens | Common Cactus-finch | Emberizidae | no | no_fam | 7 | 42 |
| Junco_phaeonotus | Yellow-eyed Junco | Emberizidae | no | no_fam | 4 | 33 |
| Spizella_breweri | Brewer's Sparrow | Emberizidae | no | no_fam | 0 | 72 |
| Nigrita_canicapillus | Grey-headed Negrofinch | Estrildidae | no | family | unknown | 10 |
| Falco_biarmicus | Lanner Falcon | Falconidae | no | no_fam | 0 | 254 |
| Falco_mexicanus | Prairie Falcon | Falconidae | no | no_fam | 0 | 249 |
| Falco_naumanni | Lesser Kestrel | Falconidae | no | no_fam | unknown | 393 |
| Falco_sparverius | American Kestrel | Falconidae | no | no_fam | 10 | 730 |
| Falco_subbuteo | Eurasian Hobby | Falconidae | no | unknown | unknown | 562 |
| Falco_tinnunculus | Common Kestrel | Falconidae | no | no_fam | unknown | 1546 |
| Carduelis_flammea | Common Redpoll | Fringillidae | no | no_fam | 0 | 161 |
| Carduelis_hornemanni | Hoary Redpoll | Fringillidae | no | no_fam | unknown | 53 |
| Carduelis_lawrencei | Lawrence's Goldfinch | Fringillidae | no | no_fam | unknown | 15 |
| Carpodacus_erythrinus | Common Rosefinch | Fringillidae | no | no_fam | unknown | 297 |
| Coccothraustes_vespertinus | Evening Grosbeak | Fringillidae | no | family | unknown | 146 |
| Hemignathus_virens | Common Amakihi | Fringillidae | yes | no_fam | 40 | 28 |
| Loxia_curvirostra | Red Crossbill | Fringillidae | no | no_fam | 45 | 499 |
| Loxia_scotica | Scottish Crossbill | Fringillidae | no | unknown | unknown | 22 |
| Haematopus_palliatus | American Oystercatcher | Haematopodidae | no | family | 135 | 70 |
| Hirundo_atrocaerulea | Blue Swallow | Hirundinidae | no | no_fam | unknown | 32 |
| Hirundo_rustica | Barn Swallow | Hirundinidae | no | no_fam | 8.7 | 1702 |
| Progne_tapera | Brown-chested Martin | Hirundinidae | no | no_fam | unknown | 8 |
| Stelgidopteryx_serripennis | Northern Rough-winged Swallow | Hirundinidae | no | no_fam | 10 | 13 |
| Tachycineta_bicolor | Tree Swallow | Hirundinidae | yes | no_fam | 0 | 541 |
| Tachycineta_thalassina | Violet-green Swallow | Hirundinidae | no | no_fam | unknown | 53 |
| Icterus_spurius | Orchard Oriole | Icteridae | no | no_fam | 35 | 53 |
| Lanius_collurio | Red-backed Shrike | Laniidae | no | no_fam | 6 | 658 |
| Sterna_dougallii | Roseate Tern | Laridae | no | family | unknown | 379 |
| Sterna_fuscata | Sooty Tern | Laridae | no | family | unknown | 302 |
| Sterna_hirundo | Common Tern | Laridae | no | family | unknown | 1517 |
| Sterna_paradisaea | Arctic Tern | Laridae | no | unknown | unknown | 639 |
| Dryoscopus_cubla | Black-backed Puffback | Malaconotidae | no | family | 279 | 15 |
| Anthochaera_carunculata | Red Wattlebird | Meliphagidae | no | no_fam | unknown | 61 |
| Lichenostomus_chrysops | Yellow-faced Honeyeater | Meliphagidae | no | no_fam | 0 | 22 |
| Lichenostomus_fuscus | Fuscous Honeyeater | Meliphagidae | no | no_fam | 30 | 3 |
| Lichenostomus_ornatus | Yellow-plumed Honeyeater | Meliphagidae | no | no_fam | 10 | 3 |
| Meliphaga_albilineata | White-lined Honeyeater | Meliphagidae | no | no_fam | unknown | 8 |
| Phylidonyris_novaehollandiae | New Holland Honeyeater | Meliphagidae | no | family | 90 | 81 |
| Grallina_cyanoleuca | Magpie-lark | Monarchidae | no | family | 159 | 58 |
| Anthus_pratensis | Meadow Pipit | Motacillidae | no | no_fam | 8 | 283 |
| Anthus_trivialis | Tree Pipit | Motacillidae | no | no_fam | 0 | 153 |
| Motacilla_capensis | Cape Wagtail | Motacillidae | no | no_fam | 25 | 17 |
| Motacilla_cinerea | Grey Wagtail | Motacillidae | yes | no_fam | 3.5 | 349 |
| Motacilla_flaviventris | Madagascar Wagtail | Motacillidae | no | unknown | unknown | 4 |
| Erithacus_rubecula | European Robin | Muscicapidae | yes | no_fam | 10 | 734 |
| Ficedula_albicollis | Collared Flycatcher | Muscicapidae | yes | no_fam | 4 | 422 |
| Ficedula_hypoleuca | European Pied Flycatcher | Muscicapidae | no | no_fam | 0 | 1296 |
| Monticola_saxatilis | Rufous-tailed Rock-thrush | Muscicapidae | no | no_fam | 0 | 99 |
| Oenanthe_leucopyga | White-tailed Wheatear | Muscicapidae | no | no_fam | 39 | 25 |
| Oenanthe_oenanthe | Northern Wheatear | Muscicapidae | no | no_fam | 16 | 399 |
| Saxicola_rubetra | Whinchat | Muscicapidae | no | no_fam | 12.5 | 232 |
| Nectarinia_zeylonica | Purple-rumped Sunbird | Nectariniidae | no | unknown | unknown | unknown |
| Oriolus_oriolus | Eurasian Golden Oriole | Oriolidae | no | unknown | unknown | 226 |
| Parus_caeruleus | Blue Tit | Paridae | yes | no_fam | unknown | 1339 |
| Parus_carolinensis | Carolina Chickadee | Paridae | no | no_fam | 40 | 139 |
| Parus_lugubris | Sombre Tit | Paridae | no | no_fam | unknown | unknown |
| Dendroica_discolor | Prairie Warbler | Parulidae | no | no_fam | 0 | 102 |
| Dendroica_striata | Blackpoll Warbler | Parulidae | no | no_fam | 0 | 103 |
| Leucopeza_semperi | Semper's Warbler | Parulidae | no | unknown | unknown | 3 |
| Vermivora_pinus | Blue-winged Warbler | Parulidae | no | no_fam | unknown | 135 |
| Wilsonia_canadensis | Canada Warbler | Parulidae | no | no_fam | 4 | 50 |
| Petroica_phoenicea | Flame Robin | Petroicidae | no | no_fam | 10 | 40 |
| Phalacrocorax_pelagicus | Pelagic Cormorant | Phalacrocoracidae | no | no_fam | unknown | 77 |
| Dendrocopos_kizuki | Pygmy Woodpecker | Picidae | no | family | unknown | 15 |
| Dendrocopos_major | Great Spotted Woodpecker | Picidae | no | unknown | unknown | 321 |
| Dendrocopos_medius | Middle Spotted Woodpecker | Picidae | no | unknown | unknown | 147 |
| Melanerpes_lewis | Lewis's Woodpecker | Picidae | no | no_fam | unknown | 42 |
| Picus_canus | Grey-faced Woodpecker | Picidae | no | family | unknown | 170 |
| Sphyrapicus_ruber | Red-breasted Sapsucker | Picidae | no | unknown | unknown | 21 |
| Sphyrapicus_thyroideus | Williamson's Sapsucker | Picidae | no | no_fam | 10 | 38 |
| Foudia_rubra | Mauritius Fody | Ploceidae | no | no_fam | 15.5 | 13 |
| Foudia_sechellarum | Seychelles Fody | Ploceidae | no | unknown | unknown | unknown |
| Aechmophorus_occidentalis | Western Grebe | Podicipedidae | no | family | unknown | 204 |
| Podiceps_auritus | Horned Grebe | Podicipedidae | no | no_fam | 25 | 236 |
| Podiceps_cristatus | Great Crested Grebe | Podicipedidae | no | family | unknown | 972 |
| Podilymbus_podiceps | Pied-billed Grebe | Podicipedidae | no | family | 100 | 173 |
| Poliocephalus_rufopectus | New Zealand Grebe | Podicipedidae | no | family | 50 | 7 |
| Rollandia_microptera | Titicaca Grebe | Podicipedidae | no | no_fam | unknown | 2 |
| Rollandia_rolland | White-tufted Grebe | Podicipedidae | no | no_fam | unknown | 18 |
| Tachybaptus_novaehollandiae | Australasian Grebe | Podicipedidae | no | family | unknown | 25 |
| Tachybaptus_ruficollis | Little Grebe | Podicipedidae | no | no_fam | unknown | 340 |
| Polioptila_californica | California Gnatcatcher | Polioptilidae | no | no_fam | 0 | 65 |
| Cyanoramphus_novaezelandiae | Red-fronted Parakeet | Psittacidae | no | family | unknown | 62 |
| Platycercus_elegans | Crimson Rosella | Psittacidae | no | no_fam | 21 | 72 |
| Rallina_tricolor | Red-necked Crake | Rallidae | no | no_fam | 0 | 22 |
| Rhipidura_hyperythra | Chestnut-bellied Fantail | Rhipiduridae | no | unknown | unknown | 2 |
| Pygoscelis_adeliae | Adelie Penguin | Spheniscidae | no | no_fam | 0 | 822 |
| Stercorarius_parasiticus | Parasitic Jaeger | Stercorariidae | no | no_fam | unknown | 425 |
| Asio_otus | Long-eared Owl | Strigidae | no | no_fam | 0 | 1129 |
| Athene_cunicularia | Burrowing Owl | Strigidae | no | no_fam | 10 | 523 |
| Bubo_lacteus | Giant Eagle-owl | Strigidae | no | coop_families | 600 | 66 |
| Megascops_asio | Eastern Screech-owl | Strigidae | no | no_fam | unknown | 312 |
| Strix_nebulosa | Great Grey Owl | Strigidae | no | family | unknown | 280 |
| Cinnyricinclus_leucogaster | Violet-backed Starling | Sturnidae | no | family | unknown | 26 |
| Sturnus_vulgaris | Common Starling | Sturnidae | yes | no_fam | 10 | 2 |
| Phylloscopus_bonelli | Bonelli's Warbler | Sylviidae | no | unknown | unknown | 123 |
| Phylloscopus_trochilus | Willow Warbler | Sylviidae | no | no_fam | 16.5 | 618 |
| Thryothorus_leucotis | Buff-breasted Wren | Troglodytidae | no | family | 244 | 14 |
| Thryothorus_pleurostictus | Banded Wren | Troglodytidae | no | family | unknown | 15 |
| Troglodytes_aedon | House Wren | Troglodytidae | yes | family | 158.5 | 356 |
| Sialia_currucoides | Mountain Bluebird | Turdidae | no | no_fam | 35 | 207 |
| Sialia_sialis | Eastern Bluebird | Turdidae | yes | family | 275.5 | 320 |
| Hirundinea_ferruginea | Cliff Flycatcher | Tyrannidae | no | family | unknown | 6 |
| Zosterops_japonicus | Japanese White-eye | Zosteropidae | no | no_fam | 2.5 | 17 |

**References**

1. Cockburn A. Prevalence of different modes of parental care in birds. Proceedings of the Royal Society B-Biological Sciences. 2006;273(1592):1375-83. doi:10.1098/rspb.2005.3458.

2. Price T, Millington S, Grant P. Helping at the nest in Darwin's finches as misdirected parental care. The Auk. 1983:192-4.

3. Jetz W, Thomas GH, Joy JB, Hartmann K, Mooers AO. The global diversity of birds in space and time. Nature. 2012;491(7424):444-8. doi:10.1038/nature11631.

4. Drobniak SM, Wagner G, Mourocq E, Griesser M. Family living: an overlooked but pivotal social system to understand the evolution of cooperative breeding. Behavioral Ecology. 2015;26(3):805-11. doi: 10.1093/beheco/arv015.

5. Valcu M, Dale J, Griesser M, Nakagawa S, Kempenaers B. Global gradients of avian longevity support the classic evolutionary theory of ageing. Ecography. 2014;37(10):930-8. doi:10.1111/ecog.00929.
