# Supplementary material for: Occasional cooperative breeding in birds and the robustness of comparative analyses concerning the evolution of cooperative breeding
Source: Zoological Lett. 2016 Mar 28;2:7. doi: 10.1186/s40851-016-0041-8 (PMC4810505; doi:10.1186/s40851-016-0041-8)
Supplement: Additional file 2: — List of species that have been observed to engage in misdirected parental care (i.e., feeding young at the nest of another species), excluding cases where species shared nests. (DOCX 30 kb) [file 40851_2016_41_MOESM2_ESM.docx]

Supplement Table 2. List of species that have been observed to engage in misdirected parental care (i.e., feed young at the nest of another species), excluding cases where species shared nests (sorted by taxonomic family). Taxonomy follows Jetz et al. [[1](#_ENREF_1)], data on occasional cooperative breeding were obtained from Cockburn [[2](#_ENREF_2)], parental care mode and the time offspring remain with their parents beyond independency (family time) follows Drobniak et al. [[3](#_ENREF_3)]. Non-family-living species where offspring disperse soon after independency (no_fam), family-living species where offspring remain well beyond independency with their parents (family), cooperatively breeding, family-living species where offspring remain with their parents and help raising younger siblings (coop_family).

| scientific name | common name | taxonomic family | N observations | occasional cooperative breeder | parental care mode | family time | reference |
| --- | --- | --- | --- | --- | --- | --- | --- |
| *Accipiter_gentilis* | Northern Goshawk | *Accipitridae* | 1 | no | no_fam | 0 | [[4](#_ENREF_4)] |
| *Aegithalos_caudatus* | Long-tailed Tit | *Aegithalidae* | 1 | no | coop_families | unknown | [[4](#_ENREF_4)] |
| *Cardinalis_cardinalis* | Northern Cardinal | *Cardinalidae* | 2 | yes | no_fam | 0 | [[4](#_ENREF_4)] |
| *Piranga_olivacea* | Scarlet Tanager | *Cardinalidae* | 1 | no | no_fam | 0 | [[4](#_ENREF_4)] |
| *Prinia_maculosa* | Karoo Prinia | *Cisticolidae* | 1 | no | no_fam | 0 | [[4](#_ENREF_4)] |
| *Pipilo_erythrophthalmus* | [Eastern Towhee](http://www.allaboutbirds.org/guide/Eastern_Towhee/id) | *Emberizidae* | 2 | no | no_fam | 0 | [[5](#_ENREF_5)] |
| *Ammodramus_maritimus* | Seaside Sparrow | *Emberizidae* | 1 | no | no_fam | 10 | [[4](#_ENREF_4)] |
| *Junco_hyemalis* | Dark-eyed Junco | *Emberizidae* | 1 | no | no_fam | 1 | [[4](#_ENREF_4)] |
| *Melospiza_melodia* | Song Sparrow | *Emberizidae* | 3 | no | no_fam | 29 | [[4](#_ENREF_4)] |
| *Pipilo_crissalis* | California Towhee | *Emberizidae* | 1 | no | no_fam | 20 | [[4](#_ENREF_4)] |
| *Spizella_pusilla* | Field Sparrow | *Emberizidae* | 1 | no | no_fam | 0 | [[4](#_ENREF_4)] |
| *Hemignathus_virens* | Common Amakihi | *Fringillidae* | 1 | yes | no_fam | 40 | [[6](#_ENREF_6)] |
| *Carpodacus_mexicanus* | House Finch | *Fringillidae* | 1 | no | no_fam | 0 | [[4](#_ENREF_4)] |
| *Fringilla_coelebs* | Eurasian Chaffinch | *Fringillidae* | 1 | no | no_fam | 24 | [[4](#_ENREF_4)] |
| *Gavia_arctica* | Arctic Loon | *Gaviidae* | 1 | no | no_fam | 15 | [[4](#_ENREF_4)] |
| *Tachycineta_bicolor* | Tree Swallow | *Hirundinidae* | 1 | yes | no_fam | 0 | [[4](#_ENREF_4)] |
| *Sterna_caspia* | Caspian Tern | *Laridae* | 1 | no | family | 90 | [[7](#_ENREF_7)] |
| *Dumetella_carolinensis* | Grey Catbird | *Mimidae* | 1 | no | no_fam | 13 | [[4](#_ENREF_4)] |
| *Toxostoma_rufum* | Brown Thrasher | *Mimidae* | 1 | no | no_fam | 45 | [[4](#_ENREF_4)] |
| *Motacilla_cinerea* | Grey Wagtail | *Motacillidae* | 2 | yes | no_fam | 3.5 | [[8](#_ENREF_8)] |
| *Ficedula_albicollis* | Collared Flycatcher | *Muscicapidae* | 1 | yes | no_fam | 4 | [[9](#_ENREF_9)] |
| *Erithacus_rubecula* | European Robin | *Muscicapidae* | 2 | yes | no_fam | 10 | [[4](#_ENREF_4)] |
| *Muscicapa_striata* | Spotted Flycatcher | *Muscicapidae* | 1 | no | no_fam | 12 | [[4](#_ENREF_4)] |
| *Arachnothera_longirostra* | Little Spiderhunter | *Nectariniidae* | 1 | no | unknown | unknown | [[10](#_ENREF_10)] |
| *Parus_minor* | Japanese Great Tit | *Paridae* | 1 | no | no_fam | unknown | this study |
| *Parus_caeruleus* | Blue Tit | *Paridae* | 2 | yes | family | unknown | [[4](#_ENREF_4)] |
| *Parus_gambeli* | Mountain Chickadee | *Paridae* | 1 | no | no_fam | 3 | [[4](#_ENREF_4)] |
| *Helmitheros_vermivorum* | Worm-eating Warbler | *Parulidae* | 2 | no | no_fam | unknown | [[4](#_ENREF_4)] |
| *Mniotilta_varia* | Black-and-white Warbler | *Parulidae* | 1 | no | no_fam | unknown | [[4](#_ENREF_4)] |
| *Passer_domesticus* | House Sparrow | *Passeridae* | 3 | no | no_fam | 7 | [[4](#_ENREF_4)] |
| *Petroica_australis* | New Zealand Robin | *Petroicidae* | 1 | no | no_fam | 21 | [[11](#_ENREF_11)] |
| *Picoides_villosus* | Hairy Woodpecker | *Picidae* | 2 | no | no_fam | 2 | [[12](#_ENREF_12)] |
| *Melanerpes_carolinus* | Red-bellied Woodpecker | *Picidae* | 1 | no | no_fam | 0 | [[4](#_ENREF_4)] |
| *Picoides_dorsalis* | American Three-toed Woodpecker | *Picidae* | 1 | no | no_fam | 40 | [[4](#_ENREF_4)] |
| *Polioptila_plumbea* | Tropical Gnatcatcher | *Polioptilidae* | 1 | no | unknown | unknown | [[4](#_ENREF_4)] |
| *Sitta_europaea* | Wood Nuthatch | *Sittidae* | 2 | no | no_fam | 4 | [[4](#_ENREF_4)] |
| *Sitta_pygmaea* | Pygmy Nuthatch | *Sittidae* | 1 | no | coop_families | 366 | [[4](#_ENREF_4)] |
| *Aptenodytes_patagonicus* | King Penguin | *Spheniscidae* | 1 | no | no_fam | 0 | [[13](#_ENREF_13)] |
| *Megascops_sp* | Screech Owl | *Strigidae* | 1 | no | no_fam | 25 | [[4](#_ENREF_4)] |
| *Sturnus_vulgaris* | Common Starling | *Sturnidae* | 4 | yes | no_fam | 10 | [[4](#_ENREF_4)] |
| *Thryothorus_ludovicianus* | Carolina Wren | *Troglodytidae* | 2 | no | no_fam | 4 | [[4](#_ENREF_4)] |
| *Troglodytes_aedon* | House Wren | *Troglodytidae* | 2 | yes | family | 158.5 | [[4](#_ENREF_4)] |
| *Troglodytes_troglodytes* | Winter Wren | *Troglodytidae* | 3 | no | no_fam | 3 | [[4](#_ENREF_4)] |
| *Hylocichla_mustelina* | Wood Thrush | *Turdidae* | 1 | no | no_fam | 7.3 | [[14](#_ENREF_14)] |
| *Turdus_migratorius* | American Robin | *Turdidae* | 3 | no | no_fam | 16 | [[15](#_ENREF_15), [4](#_ENREF_4)] |
| *Catharus_ustulatus* | Swainson's Thrush | *Turdidae* | 1 | no | no_fam | unknown | [[4](#_ENREF_4)] |
| *Sialia_sialis* | Eastern Bluebird | *Turdidae* | 3 | yes | family | 275.5 | [[4](#_ENREF_4)] |
| *Turdus_merula* | Eurasian Blackbird | *Turdidae* | 2 | no | no_fam | 0 | [[4](#_ENREF_4)] |
| *Contopus_virens* | Eastern Wood-pewee | *Tyrannidae* | 1 | no | no_fam | unknown | [[4](#_ENREF_4)] |
| *Empidonax_minimus* | Least Flycatcher | *Tyrannidae* | 1 | no | no_fam | 12 | [[4](#_ENREF_4)] |
| *Tyrannus_forficatus* | Scissor-tailed Flycatcher | *Tyrannidae* | 1 | no | no_fam | unknown | [[4](#_ENREF_4)] |

**References**

1. Jetz W, Thomas GH, Joy JB, Hartmann K, Mooers AO. The global diversity of birds in space and time. Nature. 2012;491(7424):444-8. doi:10.1038/nature11631.

2. Cockburn A. Prevalence of different modes of parental care in birds. Proceedings of the Royal Society B-Biological Sciences. 2006;273(1592):1375-83. doi:10.1098/rspb.2005.3458.

3. Drobniak SM, Wagner G, Mourocq E, Griesser M. Family living: an overlooked but pivotal social system to understand the evolution of cooperative breeding. Behavioral Ecology. 2015;26(3):805-11. doi: 10.1093/beheco/arv015.

4. Shy MM. Interspecific feeding among birds: a review. Journal of Field Ornithology. 1982:370-93.

5. Schaeffer KM, Brown WP, Gregory Shriver W. Misdirected parental care by a male Eastern Towhee at a Wood Thrush nest. The Wilson Journal of Ornithology. 2009;121(2):427-9.

6. Farmer C, Frederick BA, Banko PC, Stephens RM, Snow CW. Palila (Loxioides bailleui) fledgling fed by Hawai'i'Amakihi (Hemignathus virens). The Wilson Journal of Ornithology. 2008;120(2):416-8.

7. Oswald SA, Wails CN, Morey BE, Arnold JM. Caspian Terns (Hydroprogne caspia) Fledge a Ring-billed Gull (Larus delawarensis) Chick: Successful Waterbird Adoption Across Taxonomic Families. Waterbirds. 2013;36(3):385-9.

8. Yoerg SI, O'Halloran J. Dipper nestlings fed by a Gray Wagtail. The Auk. 1991:427-9.

9. Kristin A. Interspecific feeding at bird nests: Ficedula albicollis as a helper at the nest of Turdus philomelos. Tichodroma. 2009;21:98-101.

10. Pierce AJ. Interspecific feeding of a White-eye fledgling by a Little Spiderhunter. Natural History. 2005;5(1):41.

11. Masuda BM. Interspecific feeding of South Island tomtit (Petroica macrocephala macrocephala) nestlings and fledglings by a male Stewart Island robin (P. australis rakiura). Notornis. 2011;58:95-7.

12. Pranty B. Hairy Woodpeckers (Picoides villosus) feed Downy Woodpecker (P. pubescens) nestlings. Florida Field Naturalist. 2010;38(2):71-2.

13. Oosthuizen WC, de Bruyn PN. King penguin brooding and defending a sub-Antarctic skua chick. Polar biology. 2009;32(2):303-5.

14. Halley MR, Heckscher CM. Interspecific Parental Care by a Wood Thrush (Hylocichla mustelina) at a Nest of the Veery (Catharus fuscescens). The Wilson Journal of Ornithology. 2013;125(4):823-8.

15. Mcnair DB, Ducky B. Interspecific feeding among some oscines. Chat. 1991;55:9-11.
